# Preoperative anxiety and its association with patients’ desire for support - an observational study in adults

**DOI:** 10.1186/s12871-021-01361-2

**Published:** 2021-05-17

**Authors:** Stefan Salzmann, Stephen Rienmüller, Stefan Kampmann, Frank Euteneuer, Dirk Rüsch

**Affiliations:** 1 Department of Clinical Psychology and Psychotherapy, Philipps-University Marburg, Gutenbergstrasse 18, 35032 Marburg, Germany; 2 Philipps-University Marburg, Biegenstraße 10, 35037 Marburg, Germany; 3 Department of Psychology, Clinical Psychology and Psychotherapy, Medical School Berlin, Calandrellistrasse 1-9, 12247 Berlin, Germany; 4 Department of Anesthesia and Intensive Care, University Hospital Giessen-Marburg (Marburg Campus), Baldingerstrasse, 35043 Marburg, Germany

**Keywords:** Elective surgery, Preoperative anxiety, Amsterdam preoperative anxiety and information scale (APAIS), Emotional distress, Desire for support

## Abstract

**Background:**

Preoperative anxiety is prevalent and has harmful effects on postoperative outcomes. However, to date, it is still unclear (i) to what extent patients perceive preoperative anxiety as emotionally distressful, (ii) whether patients would welcome support from anesthesiologists in coping with their anxiety, and (iii) whether anxiety scores are useful for everyday clinical practice to determine patients’ need for support.

**Methods:**

1082 patients scheduled to undergo elective procedures under general anesthesia were eligible for this cross-sectional study carried out at a university hospital.

Preoperative anxiety, resulting in emotional distress, and patients’ desire for anesthesiologists’ support in coping with their anxiety were assessed dichotomously (no vs. yes) and analyzed descriptively. The intensity of anxiety was evaluated using the Amsterdam Preoperative Anxiety and Information Scale (range 4–20). Associations between the intensity of anxiety and the resulting desire for support were analyzed using logistic regression. Receiver operating characteristic analyses were performed to identify anxiety levels that best predict desire for support.

**Results:**

Among the 1000 (537 female; *M* (SD) 57 (18) years) subjects evaluated, 493 (318 (65 %) female) reported anxiety. Anxiety was associated with emotional distress in 320 (65 %) and desire for support in 291 (59 %) patients. Increased preoperative anxiety levels were associated with higher rates of desire for support (B= 0.270; odds ratio 1.31 [95 % CI 1.22–1.41]). An anxiety score > 9 was best to predict a desire for support (sensitivity 0.861, specificity 0.724). However, desire for support was even present in some patients with lowest anxiety scores (5 or 6).

**Conclusions:**

All patients undergoing surgery should be screened for preoperative anxiety and the resulting desire for support to be able to determine who would welcome support. Anxiety scoring tools do not seem to be useful to identify these patients. By helping patients experience less preoperative anxiety, anesthesiologists may not only reduce patients’ emotional distress but also have a positive impact on postoperative outcome.

**Trial registration:**

German Clinical Trials Register (DRKS 00013319, 23 November 2017).

**Supplementary Information:**

The online version contains supplementary material available at 10.1186/s12871-021-01361-2.

## Introduction

Preoperative anxiety is prevalent in patients undergoing elective surgery [1–4]. It is associated with increased morbidity and mortality in the postoperative period [5–7]. Besides reporting on its prevalence, many studies have also presented mean intensities of preoperative anxiety [3, 4, 8–14]. However, associations between intensities of preoperative anxiety and resulting emotional distress as well as the desire for support from anesthesiologists in coping with their anxiety have not been studied yet. A more thorough understanding of the associations between perceived preoperative anxiety and resulting emotional distress as well as patients’ need for support may help decrease patients’ emotional distress and improve postoperative outcomes.

Patients experiencing preoperative anxiety have often been divided into those with high and low anxiety [4, 9, 13, 14], assuming that the former group of patients that has commonly been referred to as “anxiety cases” would benefit from more attention. The continuous variable “preoperative anxiety” has been applied as a dichotomous variable using certain cutoff-scores (e.g., the total anxiety score of the Amsterdam Preoperative Anxiety and Information Scale (APAIS-A-T) > 10) to easily distinguish between anxiety cases and patients with low anxiety [9]. Moerman and colleagues suggested that future research should determine the usefulness of distinguishing between anxiety cases and non-anxiety cases for clinical practice [9]. Results of a recent study give rise to question the clinical benefit of such a dichotomization [15].

The main objective of this study was to examine whether and if so, to what extent patients perceive preoperative anxiety as emotionally distressful or unsettling (emotionally distressful/unsettling will also be referred to as “negative emotional impact” throughout the paper) and whether patients would welcome anesthesiologists’ support in coping with their preoperative anxiety. The underlying hypothesis for this objective was that most patients who report preoperative anxiety (no vs. yes) experience a negative emotional impact and would welcome support in coping with their anxiety. The main secondary aim of this study was to examine the associations between the intensity of a patient’s preoperative anxiety, the resulting negative emotional impact, and the desire for assistance in coping with their preoperative anxiety. The underlying hypothesis for this secondary objective was that increasing levels of preoperative anxiety are associated with higher rates of perceived negative emotional impact (no vs. yes) and increased rates of desire for support in coping with anxiety (no vs. yes). Other secondary aims were to investigate whether there is a threshold anxiety level that identifies all patients who (a) do not perceive preoperative anxiety as emotionally distressful or unsettling and who (b) would not welcome anesthesiologists’ assistance in coping with their preoperative anxiety. Secondary aims also included studying the predictive value of identifying patients with high anxiety (“anxiety cases” defined by a total anxiety score (APAIS-A-T) > 10) [9] regarding a negative emotional impact and the resulting desire for anesthesiologists’ support to cope with their preoperative anxiety.

## Methods

This observational study was part of a cross-sectional survey carried out at Marburg University Hospital investigating various aspects related to preoperative anxiety.

### Ethics approval and study registration

 The survey including this study was approved by the local ethics committee (Ethics committee of the Medical Faculty of Marburg University, approval number 134/17, dated 10 October 2017). It was registered with the German Clinical Trials Register (DRKS 00013319, 23 November 2017) and conducted in accordance with the most recent version of the Declaration of Helsinki (2013).

### Consent to participation

 Informed consent was obtained from all participants. Before completing the questionnaire, participants also learned that they had the right to abandon the completion of the questionnaire at any time without giving any reason.

### Inclusion criteria

Adult patients (≥ 18 years) scheduled to undergo any non-emergency procedure under general anesthesia were eligible to participate in this survey.

### Exclusion criteria

Patients undergoing procedures that could also be performed under regional anesthesia only were excluded from this survey. Further exclusion criteria were: illiteracy, insufficient knowledge of the German language, and visual impairments, which resulted in an inability to complete the questionnaire.

### Data collection

Patient recruitment for this survey took place at the pre-anesthetic evaluation clinic of Marburg University Hospital in patients waiting for their preoperative face-to-face assessment with a physician of the Department of Anesthesia and Intensive Care. During completion of the paper-pencil questionnaire, which took on average less than 10 min, a member of the study team was present to answer any questions patients might have had.

### Questionnaire

The questionnaire consisted of the following seven parts:

- Part 1 asked for sociodemographic variables, the scheduled procedure, the surgical consent, and previous surgeries.
- Part 2 assessed anxiety about surgery and/or anesthesia (no/yes). Patients who reported anxiety were requested to answer (a) whether the reported anxiety was related to surgery, anesthesia, or both, (b) whether the anxiety was perceived as emotionally distressful or as unsettling (no/yes), and (c) whether they would welcome to receive anesthesiologists’ assistance or support in coping with their anxiety (no/yes).
- Part 3 contained two numeric rating scales (NRS) ranging from 0 (no anxiety) to 10 (extreme anxiety) to measure anxiety related to anesthesia and surgery separately.
- Part 4 contained a German version (Additional file 1) of the Amsterdam Preoperative Anxiety and Information Scale (APAIS) [9] (Additional file 2). The APAIS has six items (statements) to assess the level of patients’ anxiety about anesthesia (APAIS-A-An, two items), their anxiety about surgery (APAIS-A-Su, two items), and their need for information concerning anesthesia and surgery (APAIS-I-T, two items). Participants were asked to indicate the applicability of the statements on a 1 (not at all) to 5 (extremely) Likert scale. Total preoperative anxiety (APAIS-A-T, four items) is the sum of anxiety about anesthesia and anxiety about surgery (APAIS-A-An plus APAIS-A-Su, maximal score = 20). The APAIS has been described in extenso and validated in many countries. Versions exist in different languages [9, 16–20] including German [11]. Results of a previous survey in more than 3.000 patients showed that the reliability (Cronbach’s α) of the four anxiety items (“anxiety scale”) and of the two information items (“information scale”) were 0.87 and 0.74, respectively [4]. Given the strong evidence concerning the validity and reliability of the APAIS and its common use in numerous studies, it can be considered the gold standard to measure preoperative anxiety.
- Part 5 addressed those survey participants who reported anxiety (in part 2 of the questionnaire), how much they sensed this anxiety about anesthesia and surgery to be emotionally distressful or unsettling using numeric rating scales with a range from 0 (not emotionally distressful/unsettling) to 10 (extremely emotionally distressful/unsettling).

Parts 6 and 7 of the questionnaire are not relevant for this study and, therefore, not described in this paper.

### Sample size

Previous results of the authors [4] and findings of other studies [9, 14] suggest that about one third of patients have high preoperative anxiety (i.e., APAIS-A-T > 10). Based on these findings, we calculated the sample size that would allow detecting even small effects/correlations regarding associations between preoperative anxiety and perceived emotional distress and desire for support in patients with low and high preoperative anxiety: Accordingly, 1000 patients (including 300 patients expected to have high anxiety) were required to be enrolled in this study.

### Statistical analyses

Answers of completed questionnaires were transferred to SPSS for Windows, version 26 (IBM, Chicago, Illinois), which was used to perform all statistical analyses. All analyses were conducted two-tailed. The significance level was 5 %.

Descriptive statistics were calculated for sociodemographic variables, surgery, and patient history related variables, anxiety levels, and the number of patients reporting a negative emotional impact and who reported a desire for support. Chi-square tests were analyzed to assess gender differences regarding the numbers of female and male patients primarily afraid of anesthesia or surgery or both and reporting a negative emotional impact and a desire for support. Procedures were graded depending on their extent and invasiveness as “minor”, “intermediate”, or “major” (Additional file 3), similar to the classification previously published by Caumo and colleagues [21].

Wilcoxon signed-rank tests were calculated to examine whether anxiety levels and levels of the negative emotional impact regarding surgery differed from anxiety levels and levels of the negative emotional impact regarding anesthesia when assessed with the APAIS and NRS. Logistic regression was used to evaluate associations and odds ratios between preoperative anxiety levels (APAIS) and (i) negative emotional impact (no vs. yes) and (ii) desire for support (no vs. yes) as outcomes. These analyses were conducted for patients who reported (questionnaire part 2) anxiety about the planned surgery and/or anesthesia separately and for all patients. For the analyses considering all patients, patients who responded that they did not have preoperative anxiety were coded as not to experience a negative emotional impact and not to have a desire for assistance from anesthesiologists in coping with their anxiety.

Exploratory moderator analyses were calculated using logistic regression testing for the interaction between gender and preoperative anxiety scores (APAIS) for the outcomes “negative emotional impact” (no vs. yes) and “desire for support” (no vs. yes).

Receiver operating characteristic (ROC) curves were calculated based on APAIS-A-T scores to identify the sensitivity and specificity of this instrument (APAIS) concerning (i) the negative emotional impact (no v. yes) and (ii) the desire to get assistance in coping with preoperative anxiety (no vs. yes). Youden index was calculated as a measure of optimal cut-off scores when sensitivity and specificity are considered equally important. ROC curves were also calculated to examine the utility of applying an APAIS-A-T score > 10, which was suggested to identify patients with high anxiety (anxiety cases) who (i) sense their preoperative anxiety as having a negative emotional impact and (ii) would welcome to get assistance in coping with their preoperative anxiety.

Crosstabs were calculated to demonstrate in detail the association between total preoperative anxiety (APAIS-A-T) and negative emotional impact (no vs. yes) as well as the need for support in coping with preoperative anxiety (no vs. yes) in patients who reported anxiety (questionnaire part 2).

## Results

Subjects were recruited from November 2017 to October 2018 until 1000 patients who had completed a questionnaire had been enrolled. During that time, 77 declined to participate in the survey, and five patients stopped completing the questionnaire. Characteristics of analyzed patients are presented in Table 1.

**Table 1:** Patient characteristics

Characteristics	All patients (*n* = 1000)	Anxiety (*n* = 493)	No Anxiety (*n* = 507)
Age (years), mean (SD)	56 (18.0)	56 (17.6)	57 (18.5)
Female, n (%)	537 (53.9)	318 (31.9)	219 (22.0)
Male, n (%)	459 (46.1)	171 (17.2)	288 (28.9)
Secondary school education, n (%)			
Lower secondary degree	365 (36.8)	177 (17.8)	188 (18.9)
Medium secondary degree	319 (32.1)	155 (15.6)	164 (16.5)
Upper secondary degree	293 (29.5)	151 (15.2)	142 (14.3)
Without secondary school degree	16 (1.6)	5 (0.5)	11 (1.1)
Number of previous surgeries, n (%)			
None	86 (8.6)	53 (10.8)	33 (6.5)
1–2	312 (31.3)	163 (33.1)	149 (29.4)
> 2	600 (60.1)	276 (56.0)	324 (63.9)
Time of intervention, n (%)			
Same day	29 (3.0)	17 (3.4)	12 (2.4)
Following day	285 (29.1)	145 (29.4)	140 (27.6)
Later than following day	665 (67.9)	321 (65.1)	344 (67.9)
Consent for procedure, n (%)			
No	270 (27.2)	131 (26.6)	139 (27.4)
Yes	722 (72.8)	358 (72.6)	364 (71.8)
Grade of procedure, n (%)			
Minor	571 (59.9)	264 (53.5)	307 (60.6)
Intermediate	228 (23.9)	116 (23.5)	112 (22.1)
Major	155 (16.2)	90 (18.3)	65 (12.8)
Surgical discipline, n (%)			
Ophthalmic	181 (18.2)	85 (17.2)	96 (18.9)
Gynecological	165 (16.6)	98 (19.9)	67 (13.2)
Ears, nose and throat	160 (16.2)	66 (13.4)	94 (18.5)
General	125 (12.6)	66 (13.4)	59 (11.6)
Urological	99 (10.0)	40 (8.1)	59 (11.6)
Neurosurgical	77 (7.8)	43 (8.7)	34 (6.7)
Oral and maxillofacial	69 (7.0)	28 (5.7)	41 (8.1)
Cardiac	55 (5.5)	29 (5.9)	26 (5.1)
Orthopedic	33 (3.3)	18 (3.7)	15 (3.0)
Trauma	16 (1.6)	9 (1.8)	7 (1.4)
Dermatological	12 (1.2)	5 (1.0)	7 (1.4)

*Anxiety* patients who reported anxiety (no vs. yes), *No anxiety* patients who did not report anxiety (no vs. yes), *Consent for procedure* completion of the questionnaire took place after surgical preoperative assessment (no vs. yes), *Grade of procedure* the assignment of the different procedures to the three grades of surgeries is shown in Additional File 3

### Anxiety, negative emotional impact, and desire for support

Almost half of the subjects reported preoperative anxiety, with a statistically significant higher fraction of women being affected (Table 2). In most patients, anxiety was related to surgery and anesthesia, with fewer patients being concerned about surgery or anesthesia only (Table 2). Analyses indicated a higher fraction of men being concerned about surgery only, while higher fractions of women seemed to be concerned about anesthesia alone or anesthesia and surgery combined. Nearly two-thirds of the patients who reported preoperative anxiety perceived their anxiety as emotionally distressful or unsettling and would welcome support in any form from anesthesiologists in coping with their anxiety (Table 2). These findings confirm our primary hypothesis. Although more women reported experiencing preoperative anxiety, among all subjects reporting preoperative anxiety, there were no differences concerning the ratios of male and female patients reporting a negative emotional impact and a desire for support (Table 1).

**Table 2:** Prevalences of anxiety, negative emotional impact, and desire for support

	Yes n (%)	No n (%)	*p*-value
Anxious about surgery and /or anesthesia			
All patients (*n* = 1000)	493 (49.3)	507 (50.7)	
Female (*n* = 537)	318 (59.2)	219 (40.8)	< 0.001
Male (*n* = 459)	171 (37.3)	288 (62.7)	
Gender not reported (*n* = 4)	4 (100)	0 (0)	
Anxious about anesthesia			
All patients(*n* = 1000)	71 (7.1)	n.a.	
Anxiety (*n* = 493)	71 (14.4)	n.a.	
Anxiety, female (*n* = 318)	51 (16.0)	n.a.	< 0.001
Anxiety, male (*n* = 171)	19 (11.1)	n.a.	
Anxiety, gender not reported (*n* = 1)	1 (100)	n.a.	
Anxious about surgery			
All patients (*n* = 1000)	147 (14.7)	n.a.	
Anxiety (*n* = 493)	147 (29.8)	n.a.	
Anxiety, female(*n* = 318)	79 (24.8)	n.a.	< 0.001
Anxiety, male (*n* = 171)	67 (39.2)	n.a.	
Anxiety, gender not reported (*n* = 1)	1 (100)	n.a.	
Anxious about both			
All patients (*n* = 1000)	275 (27.5)	n.a.	
Anxiety, female (*n* = 318)	188 (59.1)	n.a.	< 0.001
Anxiety, male (*n* = 171)	85 (49.7)	n.a.	
Anxiety, gender not reported (*n* = 2)	2 (100)	n.a.	
Negative emotional impact			
All patients (*n* = 1000)	320 (32.0)	n.a.	
Anxiety (*n* = 488, MD = 5)	320 (64.9)	168 (34.1)	
Anxiety, female (*n* = 315)	210 (66.7)	105 (33.3)	0.459
Anxiety, male (*n* = 169)	107 (63.3)	62 (36.7)	
Anxiety, gender not reported (*n* = 4)	3 (75.0)	1 (25.0)	
Support			
All patients (*n* = 1000)	291 (29.1)	n.a.	
Anxiety, (*n* = 478, MD = 15)	291 (59.0)	187 (37.9)	
Anxiety, female (*n* = 308)	195 (63.3)	113 (36.7)	0.155
Anxiety, male(*n* = 166)	94 (56.6)	72 (43.4)	
Anxiety, gender not reported (*n* = 4)	2 (50.0)	2 (50.0)	

*Anxiety* patients who reported anxiety (no vs. yes), *Negative emotional impact* patients who reported perceiving their preoperative anxiety as emotionally distressful or unsettling (no vs. yes), *Support* patients who reported a desire for support from anesthesiologists in coping with their preoperative anxiety (no vs. yes), *MD* missing data, *(%)* the relative frequencies given refer to different samples, the size of which is specified in parentheses following the corresponding variable in the same line, *p-value* Chi-Square test statistic for differences between male and female patients, *n.a*. not applicable

The mean surgery anxiety level was significantly higher than the mean anesthesia anxiety level (Table 3). Accordingly, the mean level of negative emotional impact caused by anxiety about surgery was significantly higher than the mean level of negative emotional impact caused by anesthesia-related anxiety (Table 3).

**Table 3:** APAIS scores and NRS scores related to anxiety and negative emotional impact

Scale	Score mean (SD)	p-value	Z-value	Cohen’s d
APAIS-A-Su	5.1 (2.3)	< 0.0001	14.7	0.46
APAIS-A-An	4.1 (2.0)			
APAIS-A-T	9.2 (3.8)			
APAIS-I-T	5.7 (2.1)			
NRS-A-Su	3.6 (2.9)	< 0.0001	10.1	0.25
NRS-A-An	2.9 (2.7)			
NRS-A-T	6.4 (5.2)			
NRS-NEI-Su	3.4 (2.9)	< 0.0001	10.9	0.25
NRS-NEI-An	2.7 (2.6)			
NRS-NEI-T	6.0 (5.2)			

*APAIS* Amsterdam preoperative anxiety and information scale, *NRS* numeric rating scale, *APAIS-A-Su* APAIS anxiety about surgery score, *APAIS-A-An* APAIS anxiety about anesthesia score, *APAIS-A-T* APAIS anxiety about anesthesia and surgery score (total APAIS anxiety score), *APAIS-I-T* APAIS need for information about anesthesia and surgery score (total APAIS information score), *NRS-A-Su* NRS anxiety about surgery score *NRS-A-An* NRS anxiety about anesthesia score *NRS-A-T* NRS anxiety about anesthesia and surgery score (total NRS anxiety score), *NRS-NEI-Su* NRS score of perceiving surgery-related anxiety as having a negative emotional impact, *NRS-NEI-An* NRS score of perceiving anesthesia-related anxiety as having a negative emotional impact *NRS-NEI-T* NRS score of perceiving total anxiety about anesthesia and surgery as having a negative emotional impact *p-value* p-values were calculated using the Wilcoxon signed-rank test for comparison of the corresponding anesthesia and surgery scores

Correlations between mean anxiety scores and the corresponding mean scores of negative emotional impact were all very high: surgery (*r* = .914, *p* < .001, *n* = 971), anesthesia (*r* = .9, *p* < .001, *n* = 972), surgery and anesthesia (*r* = .922, *p* < .001, *n* = 970).

### Associations between anxiety, negative emotional impact, and desire for support

Results of logistic regression confirmed our main secondary hypothesis: Increasing levels of preoperative anxiety (APAIS-A-T) were associated with higher rates of perceived negative emotional impact (no vs. yes: B = 0.407; SE = 0.046, OR = 1.50 [95 % CI 1.37–1.64], *p* < .001) and increased rates of desire for support in coping with anxiety (no vs. yes: B = 0.270, SE = 0.037; OR = 1.31 [95 % CI 1.22–1.41], *p* < .001) when analyzing only patients who reported preoperative anxiety (no vs. yes). Increasing preoperative anxiety levels were also associated with higher rates of perceived negative emotional impact (no vs. yes: B = 0.615; SE = 0.04, OR = 1.85 [95 % CI 1.71–2.00], *p* < .001) and increased rates of desire for support in coping with anxiety (no vs. yes: B = 0.495; SE = 0.033, OR = 1.64 [95 % CI 1.54–1.75], *p* < .001) when analyzing all patients.

Gender did not moderate the association between increasing levels of preoperative anxiety and increased rates of negative emotional impact (interaction of APAIS-A-T*gender in patients who reported preoperative anxiety: B = − 0.001; SE = 0.098; OR = 0.99 [95 % CI 0.82–1.21], *p* = .991; interaction of APAIS-A-T*gender when analyzing all patients: B = 0.036, SE = 0.083; OR = 1.04 [95 % CI 0.88–1.22], *p* = .662) or desire for support in coping with anxiety (interaction of APAIS-A-T*gender in patients who reported preoperative anxiety: B = 0.093, SE = 0.082; OR = 1.10 [95 % CI 0.93–1.29], *p* = .260; interaction of APAIS-A-T*gender when analyzing all patients: B = 0.130, SE = 0.072; OR = 1.14 [95 % CI 0.99–1.31], *p* = .072).

In few cases (6 out of 482), even the lowest anxiety levels (defined as patients who scored two at one or two of the anxiety items resulting in an APAIS-A-T score of 5 or 6) reported having a negative emotional impact (Additional file 4). This was also the case (5 out of 473) with respect to patients’ desire for anesthesiologists’ assistance in coping with their anxieties (Additional file 4).

### Prediction of negative emotional impact and desire for support

The anxiety level to predict most accurately a negative emotional impact equaled APAIS-A-T > 9 with a sensitivity of 0.899, a specificity of 0.755, and a Youden index of 0.653 when equally weighing sensitivity and specificity. The threshold to best predict patients’ desire for assistance in coping with their anxiety was also APAIS-A-T > 9 with a sensitivity of 0.861, a specificity of 0.724, and a Youden index of 0.585. The commonly used APAIS-A-T score > 10 to detect patients with high anxiety had a sensitivity of 0.774 and 0.733, a specificity of 0.857 and 0.824, and a Youden index of 0.631 and 0.556 to detect a negative emotional impact and a desire for support in coping with their anxiety, respectively.

## Discussion

This study confirms the high prevalence of preoperative anxiety in patients undergoing elective surgery. More importantly, it reveals that preoperative anxiety is associated with a negative emotional impact and desire for support in many patients (65 and 59 %, respectively). As expected, this study also shows that increased levels of preoperative anxiety are associated with higher rates of emotional distress and desire for support. However, emotional distress and desire for support is even present in some patients with lowest anxiety levels.

### Preoperative anxiety and emotional distress

Despite the high prevalence of preoperative anxiety, the question of whether patients perceive their preoperative anxiety as emotionally distressful or unsettling and whether this is associated with a desire for support from anesthesiologists is still a clinically important research gap. Therefore, the first main finding of the present study is that almost two-thirds of the patients perceive their preoperative anxiety as emotionally distressful or unsettling. The relevance of these results is corroborated by an observational study carried out in > 16.000 patients showing that anxiety was most commonly reported as the worst aspect of the surgical episode [22]. Interestingly, the intensities of the perceived negative emotional impact related to anesthesia, surgery, and both according to NRS scores all correlate strongly with the corresponding NRS anxiety scores.

### Preoperative anxiety and desire for support

The second main finding being even more important is that almost all patients who perceive their anxiety as having a negative emotional impact would welcome to receive support from anesthesiologists in coping with their anxiety. This result is novel, and it clearly illustrates the importance of preoperative anxiety to many patients. We interpret these findings as an explicit request to anesthesiologists to take care of patient’s preoperative anxiety.

### Prediction of negative emotional impact and desire for support

As expected, this study also demonstrates that increasing intensities of anxiety are associated with a significantly higher likelihood of patients perceiving their preoperative anxiety as emotionally distressful or unsettling and sensing a desire for support. Unfortunately, this apparent association between intensity of anxiety and negative emotional impact as well as desire for support does not help the clinician unambiguously predict which patient should be offered assistance in coping with their anxiety. Neither does the evaluation of the intensity of anxiety (anxiety level) in general. According to this study, there is no specific anxiety level (i.e. threshold) that reliably discriminates between all patients who desire support in coping with their preoperative anxiety and those who do not. Accordingly, even some of the patients with the lowest anxiety levels reported a negative emotional impact and a desire for support.

In this context it is interesting to realize that ROC analyses showed that the best anxiety level to predict a desire for support equaled APAIS-A-T > 9 with a sensitivity of 86 % and a specificity of 72 %. In comparison, an APAIS-A-T > 10 that was suggested by Moerman and colleagues to distinguish “anxiety cases” from “non-anxious patients” [9] had a sensitivity of 73 % only (with a specificity of 82 %) to detect patients of this study who would welcome assistance in coping with their anxiety. The existence of an anxiety score that distinguishes between patients who perceive their anxiety as emotionally distressful or unsettling and who would welcome support (from those patients who do not) would justify integrating an anxiety scoring instrument in patient history forms completed by patients before being seen by the anesthesiologist. This would help the busy and time-limited clinician to identify at a glance in what patient this issue needs to be addressed. However, in light of previous results, [4, 15], and above all findings of this study, the routine application of anxiety scoring instruments in patient history forms seems to be of limited use. For instance, in our study, 77 patients (who reported a desire for support) out of 177 patients (who reported to experience preoperative anxiety and who would be classified as non-anxiety cases according to Moerman and colleagues (APAIS-A-T < 11) [9]) would not have been identified as patients who desire support when identifying patients with a desire for support by the APAIS only. In other words, about 45 % of patients with a desire for support would have been missed when using the APAIS to identify patients who desire support.

Instead, findings of the present study suggest that all patients scheduled to undergo surgery should be screened routinely for anxiety about surgery and anesthesia as well as their desire for assistance in coping with it using dichotomous questions similar to the ones used in the present study. This would enable anesthesiologists to quickly identify patients who need attention in this respect. By analogy, if patient history forms are not used, all patients should be asked about preoperative anxiety and the desire for assistance in coping with it using dichotomous questions (no/yes). Patients who would welcome support in coping with their anxiety should be asked what kind of assistance they would prefer as patient preferences in this respect vary substantially [23].

### Gender, preoperative anxiety, negative emotional impact, and desire for support

This study is consistent with the many studies demonstrating that more women than men report experiencing preoperative anxiety [2, 8–10, 21]. However, among all patients reporting preoperative anxiety in this study, there were no differences between the ratios of male and female patients reporting a negative emotional impact and a desire for support. This means that patients experiencing preoperative anxiety are very likely to also experience a negative emotional impact and as a result to desire support in coping with their anxiety irrespective of the gender.

### Causes of preoperative anxiety

Results of the present study also demonstrate that the majority of patients reporting preoperative anxiety were anxious because of surgery and anesthesia. These findings are consistent with the results by McKenzie and colleagues. They found that in 23 %, 27 %, and 38 % of patients, respectively, anesthesia, surgery, and a combination of the two were the principal causes of concern [24]. However, in this context it is essential to realize that almost half of those who reported preoperative anxiety in the present study were anxious about surgery or anesthesia only. These findings corroborate the results of a previous study by the authors which showed that the levels of anxiety related to surgery and anesthesia differ substantially in many patients [4]. Accordingly, this finding also confirms conclusions from previous studies by the authors [4, 15] suggesting that individual patients require special attention by the surgeon or the anesthesiologist concerning their preoperative anxiety.

### Limitations

Given this single-center design of this study, results may not be valid in other regions of the world. Not all patients that would have been eligible for inclusion were contacted; instead, recruitment of patients was at random. These aspects reduce the generalizability of the study results.

## Conclusions

Results of this study emphasize the relevance of preoperative anxiety from patients’ perspective. As a consequence of these results, all patients scheduled to undergo surgery should be screened routinely for preoperative anxiety (discriminating between anxiety related to surgery and anesthesia), its negative emotional impact, and a resulting desire for support in coping with it. In this context, simple dichotomous questions (no vs. yes) may be more helpful instead of assessing anxiety levels using anxiety scoring tools. By helping patients experience less preoperative anxiety, anesthesiologists may not only reduce patients’ emotional distress but also increase chances for better postoperative outcomes. Future research should examine what anxiety alleviating interventions work best in patients who would welcome support in coping with their preoperative anxiety.

## Supplementary Information

**Additional file 1: **German version of the Amsterdam Preoperative Anxiety and Information Scale (APAIS), (Part 4 of the questionnaire). Description: Wording of the validated German translation [11] of the English version of the APAIS published by Moerman and colleagues [9].

**Additional file 2: **English version of the Amsterdam Preoperative Anxiety and Information Scale (APAIS). Wording of the English version of the APAIS published by Moerman and colleagues [9]. Items have to be rated by participants on a 1 (not at all) to 5 (extremely) Likert scale.

**Additional file 3: **Grading of procedures. Grading of procedures depending on their extent and invasiveness as “minor”, “intermediate” or “major”.

**Additional file 4: **Crosstable. Negative emotional impact and desire for support depending on intensity of anxiety.

## Data Availability

The datasets used and analyzed during the current study are available from the corresponding author on reasonable request.

## Abbreviations

APAIS: Amsterdam preoperative anxiety and information scale
APAIS-A-An: APAIS anesthesia anxiety score
APAIS-A-Su: APAIS surgery anxiety score
APAIS-A-T: APAIS total anxiety score
APAIS-I-T: APAIS total information score
NEI: Negative emotional impact
NEI-An: NEI associated with anesthesia anxiety
NEI-SU: NEI associated with surgery anxiety
NEI-T: NEI associated with total anxiety
NRS: Numeric rating scale
OR: Odds ratio
ROC: Receiver operating characteristic
SD: Standard deviation

## References

[1] Mitchell M (2010). General anaesthesia and day-case patient anxiety. J Adv Nurs..

[2] Mavridou P, Dimitriou V, Manataki A, Arnaoutoglou E, Papadopoulos G (2013). Patient’s anxiety and fear of anesthesia: effect of gender, age, education, and previous experience of anesthesia. A survey of 400 patients. J Anesth..

[3] Lee JS, Park YM, Ha KY, Cho SW, Bak GH, Kim KW (2016). Preoperative anxiety about spinal surgery under general anesthesia. Eur Spine J..

[4] Aust H, Eberhart L, Sturm T, Schuster M, Nestoriuc Y, Brehm F (2018). A cross-sectional study on preoperative anxiety in adults. J Psychosom Res..

[5] Williams JB, Alexander KP, Morin JF, Langlois Y, Noiseux N, Perrault LP (2013). Preoperative anxiety as a predictor of mortality and major morbidity in patients aged > 70 years undergoing cardiac surgery. Am J Cardiol..

[6] Tully PJ, Baker RA, Knight JL (2008). Anxiety and depression as risk factors for mortality after coronary artery bypass surgery. J Psychosom Res..

[7] Cserep Z, Losoncz E, Balog P, Szili-Torok T, Husz A, Juhasz B (2012). The impact of preoperative anxiety and education level on long-term mortality after cardiac surgery. J Cardiothorac Surg..

[8] Badner NH, Nielson WR, Munk S, Kwiatkowska C, Gelb AW (1990). Preoperative anxiety: detection and contributing factors. Can J Anaesth..

[9] Moerman N, van Dam FS, Muller MJ, Oosting H (1996). The Amsterdam Preoperative Anxiety and Information Scale (APAIS). Anesth Analg..

[10] Kindler CH, Harms C, Amsler F, Ihde-Scholl T, Scheidegger D (2000). The visual analog scale allows effective measurement of preoperative anxiety and detection of patients’ anesthetic concerns. Anesth Analg..

[11] Berth H, Petrowski K, Balck F (2007). The Amsterdam Preoperative Anxiety and Information Scale (APAIS) - the first trial of a German version. Psychosoc Med..

[12] Perks A, Chakravarti S, Manninen P (2009). Preoperative anxiety in neurosurgical patients. J Neurosurg Anesthesiol..

[13] Goebel S, Kaup L, Mehdorn HM (2011). Measuring preoperative anxiety in patients with intracranial tumors: the Amsterdam preoperative anxiety and information scale. J Neurosurg Anesthesiol..

[14] Laufenberg-Feldmann R, Kappis B (2013). Assessing preoperative anxiety using a questionnaire and clinical rating: a prospective observational study. Eur J Anaesthesiol..

[15] Eberhart L, Aust H, Schuster M, Sturm T, Gehling M, Euteneuer F (2020). Preoperative anxiety in adults - a cross-sectional study on specific fears and risk factors. BMC Psychiatry..

[16] Nishimori M, Moerman N, Fukuhara S, van Dam FS, Muller MJ, Hanaoka K (2002). Translation and validation of the Amsterdam preoperative anxiety and information scale (APAIS) for use in Japan. Qual Life Res..

[17] Boker A, Brownell L, Donen N (2002). The Amsterdam preoperative anxiety and information scale provides a simple and reliable measure of preoperative anxiety. Can J Anaesth..

[18] Maurice-Szamburski A, Loundou A, Capdevila X, Bruder N, Auquier P (2013). Validation of the French version of the Amsterdam preoperative anxiety and information scale (APAIS). Health Qual Life Outcomes..

[19] Vergara-Romero M, Morales-Asencio JM, Morales-Fernandez A, Canca-Sanchez JC, Rivas-Ruiz F, Reinaldo-Lapuerta JA (2017). Validation of the Spanish version of the Amsterdam Preoperative Anxiety and Information Scale (APAIS). Health Qual Life Outcomes..

[20] Buonanno P, Laiola A, Palumbo C, Spinelli G, Terminiello V, Servillo G (2017). Italian validation of the Amsterdam Preoperative Anxiety and Information Scale. Minerva Anestesiol..

[21] Caumo W, Schmidt AP, Schneider CN, Bergmann J, Iwamoto CW, Bandeira D (2001). Risk factors for preoperative anxiety in adults. Acta Anaesthesiol Scand..

[22] Walker EM, Bell M, Cook TM, Grocott MP, Moonesinghe SR (2016). Patient reported outcome of adult perioperative anaesthesia in the United Kingdom: a cross-sectional observational study. Br J Anaesth..

[23] Aust H, Rusch D, Schuster M, Sturm T, Brehm F, Nestoriuc Y (2016). Coping strategies in anxious surgical patients. BMC Health Serv Res..

[24] Mackenzie JW (1989). Daycase anaesthesia and anxiety. A study of anxiety profiles amongst patients attending a day bed unit. Anaesthesia..

