# Supplementary material for: Preoperative anxiety and its association with patients’ desire for support - an observational study in adults
Source: BMC Anesthesiol. 2021 May 17;21:149. doi: 10.1186/s12871-021-01361-2 (PMC8127269; doi:10.1186/s12871-021-01361-2)
Supplement: Supplementary file 1 — Additional file 1: German version of the Amsterdam Preoperative Anxiety and Information Scale (APAIS), (Part 4 of the questionnaire). Description: Wording of the validated German translation [11] of the English version of the APAIS published by Moerman and colleagues [9]. [file 12871_2021_1361_MOESM1_ESM.docx]

Preoperative anxiety and its association with patients’ desire for support - An observational study in adults

Stefan Salzmann, Stephen Rienmüller, Stefan Kampmann, Frank Euteneuer,

Dirk Rüsch

**Additional file 1 -** Amsterdam Preoperative Anxiety and Information Scale (APAIS) – German version [11]

|  | **1**  (gar nicht) | **2**  (wenig) | **3**  (mittel) | **4**  (stark) | **5**  (extrem) |
| --- | --- | --- | --- | --- | --- |
| Ich mache mir Sorgen über die Anästhesie (Narkose) |  |  |  |  |  |
| Die Anästhesie (Narkose) geht mir ständig durch den Kopf |  |  |  |  |  |
| Ich möchte so viel wie möglich über die Anästhesie (Narkose) wissen |  |  |  |  |  |
| Ich mache mir Sorgen über die Operation |  |  |  |  |  |
| Die Operation geht mir ständig durch den Kopf |  |  |  |  |  |
| Ich möchte so viel wie möglich über die Operation wissen |  |  |  |  |  |
