# Supplementary material for: Preoperative anxiety and its association with patients’ desire for support - an observational study in adults
Source: BMC Anesthesiol. 2021 May 17;21:149. doi: 10.1186/s12871-021-01361-2 (PMC8127269; doi:10.1186/s12871-021-01361-2)

Preoperative anxiety and its association with patients’ desire for support - An observational study in adults

Stefan Salzmann, Stephen Rienmüller, Stefan Kampmann, Frank Euteneuer,

Dirk Rüsch

**Additional file 2 -** Amsterdam Preoperative Anxiety and Information Scale (APAIS) – English version [9]


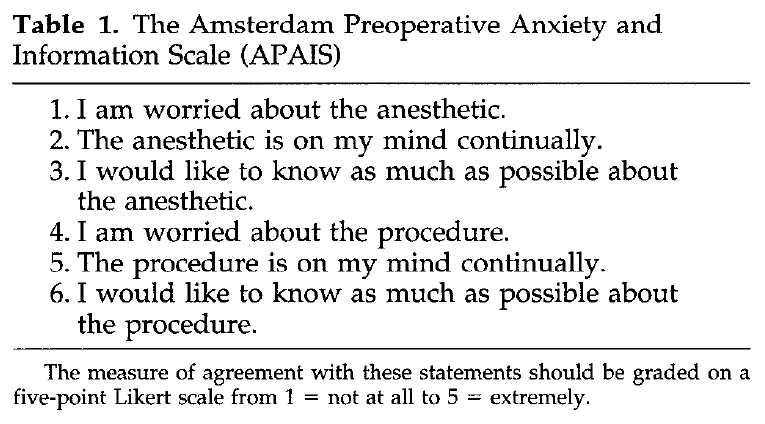

Supplement: Supplementary file 2 — Additional file 2: English version of the Amsterdam Preoperative Anxiety and Information Scale (APAIS). Wording of the English version of the APAIS published by Moerman and colleagues [9]. Items have to be rated by participants on a 1 (not at all) to 5 (extremely) Likert scale. [file 12871_2021_1361_MOESM2_ESM.docx]
