# Supplementary material for: Preoperative anxiety and its association with patients’ desire for support - an observational study in adults
Source: BMC Anesthesiol. 2021 May 17;21:149. doi: 10.1186/s12871-021-01361-2 (PMC8127269; doi:10.1186/s12871-021-01361-2)
Supplement: Supplementary file 3 — Additional file 3: Grading of procedures. Grading of procedures depending on their extent and invasiveness as “minor”, “intermediate” or “major”. [file 12871_2021_1361_MOESM3_ESM.docx]

Preoperative anxiety and its association with patients’ desire for support - An observational study in adults

Stefan Salzmann, Stephen Rienmüller, Stefan Kampmann, Frank Euteneuer,

Dirk Rüsch

**Additional file 3 -** Grading of surgical procedures

| Grade | Surgical procedure |
| --- | --- |
| Minor | Abscess incision and drainage, arthroscopic shoulder surgery (e.g. subacromial decompression, shoulder ligament reconstruction), basal cell carcinoma surgery, breast conserving therapy, breast implants removal, bursal resection, cardiac pacemaker implantation, endobronchial ultrasound with/without transbronchial needle aspiration, excision of cutaneous melanoma and sentinel lymph nodes, hematoma drainage, herniotomy (e.g. inguinal and umbilical repair), kyphoplasty, laparoscopic procedures (appendectomy, cholecystectomy, adnexal surgery for benign diseases), lymphadenectomy (cervical, axillary, inguinal), minor osteosynthesis surgery (e.g. osteosynthesis for ac-joint separation), microlaryngoscopy with biopsy, ophthalmic surgery, pacemaker generator change, parotidectomy, removal of metal implants after fracture, scar revision surgery, septoplasty/septorhinoplasty, stapedectomy, surgery for pilonidal disease, thyroidectomy, tonsillectomy, tympanoplasty, ureterorenoscopy with/without placement of ureteral stent, vacuum assisted closure, wisdom tooth extraction. |
| Intermediate | Bimaxillary osteotomy, breast reduction surgery, carotid endarterectomy, cochlear implant implantation, hysterectomy (abdominal, vaginal), mastectomy, myomectomy, neck dissection, oophorectomy, radical prostatectomy, salpingo-oophorectomy, spinal disc surgery (e.g. lumbar discectomy), spinal surgery (e.g. laminectomy, spinal fusion), tuboplasty, ureterectomy, vulvectomy. |
| Major | Adrenalectomy, bile duct surgery, cardiac surgery with sternotomy, cholecystectomy (open), cystectomy, gastrectomy, hepatectomy (partial), hysterectomy (radical), intracranial surgical procedures, major joint surgery (e.g. revision total hip replacement), lung surgery with thoracotomy, nephrectomy, pancreatectomy, rectosigmoidectomy, splenectomy. |
