# Supplementary material for: Preoperative anxiety and its association with patients’ desire for support - an observational study in adults
Source: BMC Anesthesiol. 2021 May 17;21:149. doi: 10.1186/s12871-021-01361-2 (PMC8127269; doi:10.1186/s12871-021-01361-2)
Supplement: Supplementary file 4 — Additional file 4: Crosstable. Negative emotional impact and desire for support depending on intensity of anxiety. [file 12871_2021_1361_MOESM4_ESM.docx]

Preoperative anxiety and its association with patients’ desire for support - An observational study in adults

Stefan Salzmann, Stephen Rienmüller, Stefan Kampmann, Frank Euteneuer,

Dirk Rüsch

**Additional file 4 -** Negative emotional impact and desire for support depending on intensity of anxiety

| APAIS-A-T | (n) | NEI (n) | | Support (n) | |
| --- | --- | --- | --- | --- | --- |
|  | | No | Yes | No | Yes |
| 4 | 2 | 2 | 0 | 1 | 1 |
| 5 | 7 | 4 | 3 | 6 | 1 |
| 6 | 14 | 11 | 3 | 10 | 4 |
| 7 | 13 | 7 | 6 | 7 | 6 |
| 8 | 35 | 29 | 6 | 19 | 17 |
| 9 | 33 | 19 | 14 | 21 | 11 |
| 10 | 75 | 36 | 39 | 36 | 37 |
| 11 | 50 | 23 | 27 | 24 | 23 |
| 12 | 76 | 20 | 56 | 30 | 44 |
| 13 | 46 | 7 | 39 | 17 | 29 |
| 14 | 48 | 4 | 44 | 4 | 44 |
| 15 | 20 | 3 | 17 | 3 | 16 |
| 16 | 29 | 0 | 29 | 4 | 24 |
| 17 | 8 | 0 | 8 | 0 | 8 |
| 18 | 13 | 2 | 11 | 0 | 13 |
| 19 | 2 | 0 | 2 | 0 | 2 |
| 20 | 11 | 1 | 10 | 3 | 8 |

*APAIS* Amsterdam preoperative anxiety and information scale, *APAIS-A-T* APAIS anxiety about anesthesia and surgery score (APAIS total anxiety score), *NEI* patients who reported a negative emotional impact, *Support* patients who would welcome to get support from anesthesiologists in coping with their anxiety.
